# A Frameshift Mutation in the Mg-Chelatase I Subunit Gene *OsCHLI* Is Associated with a Lethal Chlorophyll-Deficient, Yellow Seedling Phenotype in Rice

**DOI:** 10.3390/plants12152831

**Published:** 2023-07-31

**Authors:** Kyu-Chan Shim, Yuna Kang, Jun-Ho Song, Ye Jin Kim, Jae Kwang Kim, Changsoo Kim, Thomas H. Tai, Inkyu Park, Sang-Nag Ahn

**Affiliations:** 1 Department of Agronomy, College of Agriculture and Life Science, Chungnam National University, Daejeon 34134, Republic of Korea; kshim@ucdavis.edu (K.-C.S.); dkwl3120@cnu.ac.kr (Y.K.); changsookim@cnu.ac.kr (C.K.); 2 USDA-ARS Crops Pathology and Genetics Research Unit, Davis, CA 95616, USA; thomas.tai@usda.gov; 3 Department of Plant Sciences, University of California, Davis, CA 95616, USA; 4 Department of Biology, Chungbuk National University, Cheongju 28644, Republic of Korea; jhsong@cbnu.ac.kr; 5 Division of Life Sciences, College of Life Sciences and Bioengineering, Incheon National University, Incheon 22012, Republic of Korea; 201721047@inu.ac.kr (Y.J.K.); kjkpj@inu.ac.kr (J.K.K.); 6 Department of Biology and Chemistry, Changwon National University, Changwon 51140, Republic of Korea

**Keywords:** yellow seedling, magnesium chelatase, chlorophyll, carotenoid, rice

## Abstract

Chlorophyll biosynthesis is a crucial biological process in plants, and chlorophyll content is one of the most important traits in rice breeding programs. In this study, we identified a lethal, chlorophyll-deficient, yellow seedling (YS) phenotype segregating in progeny of CR5055-21, an F_2_ plant derived from a backcross between Korean *japonica* variety ‘Hwaseong’ (*Oryza sativa*) and CR5029, which is mostly Hwaseong with a small amount of *Oryza grandiglumis* chromosome segments. The segregation of the mutant phenotype was consistent with a single gene recessive mutation. Light microscopy of YS leaf cross-sections revealed loosely arranged mesophyll cells and sparse parenchyma in contrast to wildtype. In addition, transmission electron microscopy showed that chloroplasts did not develop in the mesophyll cells of the YS mutant. Quantitative trait loci (QTL)-seq analysis did not detect any significant QTL, however, examination of the individual delta-SNP index identified a 2-bp deletion (AG) in the *OsCHLI* gene, a magnesium (Mg)-chelatase subunit. A dCAPs marker was designed and genotyping of a segregating population (*n* = 275) showed that the mutant phenotype co-segregated with the marker. The 2-bp deletion was predicted to result in a frameshift mutation generating a premature termination. The truncated protein likely affects formation and function of Mg-chelatase, which consists of three different subunits that together catalyze the first committed step of chlorophyll biosynthesis. Transcriptome analysis showed that photosynthesis and carbohydrate metabolism pathways were significantly altered although expression of *OsCHLI* was not. Chlorophyll- and carotenoid-related genes were also differentially expressed in the YS mutant. Our findings demonstrated that *OsCHLI* plays an important role in leaf pigment biosynthesis and leaf structure development in rice.

## 1. Introduction

Rice is widely used as a staple food in many Asian countries, and rice production must be increased to meet the demands of the growing world population [1]. Rice plants accumulate carbohydrates through photosynthesis in leaves, and the carbohydrate pool moves to the grain [2]. Chlorophyll is a photosynthetic pigment that plays the central role in plant photosystems. As photosynthetic ability is positively correlated with chlorophyll content, many studies have been conducted on the biological processes of chlorophyll synthesis and degradation [3,4,5]. The chlorophyll biosynthesis in higher plants comprises 16 steps from glutamate to chlorophyll b with three sub-steps [6]. The first sub-step is the formation of 5-aminolevulinic acid (ALA) from glutamate followed by the development of protoporphyrin IX (Proto IX). The last step is the formation of chlorophyll b from Proto IX [7].

Carotenoids (lutein and carotenoid) and flavonoids (anthocyanins) are leaf pigments that affect leaf color in rice. In plants, carotenoids are essential pigments for optimal photosynthetic performance in photosynthetic organs and act as photo-protectors, antioxidants, color attractants, and plant hormone precursors in non-photosynthetic organs [8,9]. Pre-harvest sprouting mutants (*phs*) were identified from the T-DNA insertion mutant population and these mutants involved carotenoid precursors of ABA biosynthesis [10]. The *phs* mutants exhibited altered rice leaf color and mutation of *OsCRTISO* and *OsLCY* causes photooxidative damage to leaf photosystem II. Anthocyanin biosynthesis pathway genes, such as *OsPL*, *OsPL6*, and *PLR4,* also regulate leaf color in rice [11,12,13].

Many genes associated with chlorophyll biosynthesis have been identified using leaf color mutants derived from genetic populations or mutagenesis, and various leaf color phenotypes have been observed [14]. Most rice leaf color mutants are associated with chlorophyll metabolism [6]. One of the most common is the albino mutant, which lacks all leaf pigments (chlorophylls, carotenoids, and anthocyanins). Others that have been reported include chlorosis, thermo-color, light green, green-revertible albino, purple, white-striped, stay-green, yellow, and lethal yellow leaves [6,15,16,17,18]. A number chlorophyll-deficient yellow leaf mutants have been reported in *Arabidopsis*, barley, and rice, and most have mutations affecting Mg-chelatase and production of Mg-Proto IX [15,17,19]. 

Mg-chelatase consists of three subunits (CHLI, CHLD, and CHLH) and catalyzes the insertion of magnesium (Mg^2+^) into protoporphyrin IX (Proto IX) to form Mg-Proto IX [20,21]. Recessive *xantha-h* mutants have been identified in barley and their yellow seedlings lack Mg-chelatase activity [22]. In *Arabidopsis*, *gun4*, a chlorophyll-deficient mutant shows a lethal yellow leaf phenotype [23]. The *gun4* gene encodes a porphyrin-binding protein that regulates Mg-chelatase activity by binding to CHLH [24]. Jung et al. (2003) identified a chlorophyll-deficient *chlorina* rice mutant using a T-DNA gene-trap system [15]. A T-DNA insertion in *OsCHLH* resulted in a loss of function and caused the irregular chloroplast development [15]. More recently, a point mutation in another Mg-chelatase subunit, *OsCHLI*, was shown to result in a chlorophyll-deficient *etiolated leaf and lethal* (*ell*) phenotype in rice seedlings with heterozygous (*ELL*/*ell*) seedlings exhibiting a light-green leaf color [19]. The point mutation (G529C) in *OsCHLI* blocked the interaction of OsCHLI and OsCHLH in a yeast-two-hybrid assay [19]. The *chlorotic seedling 3* (*cs3*) chlorophyll-deficient mutant is also lethal at the seedling stage [25]. *CS3* encodes a Ycf54 domain-containing protein that functions in chlorophyll biosynthesis by negatively affecting the activity of magnesium protoporphyrin IX monomethyl ester cyclase [25]. Although many chlorophyll-deficient yellow leaf mutants have been identified and characterized, the metabolic pathways involved in these and other leaf color mutants remain poorly understood.

In this study, we identified a lethal yellow seedling (YS) mutant in the progeny of a cross between CR5029 and a Korean *japonica* rice variety ‘Hwaseong’ (*Oryza sativa*). CR5029 is an introgression line harboring *O. grandiglumis* chromosomal segments in the Hwaseong genetic background. High-performance liquid chromatography (HPLC) analysis was conducted to analyze the pigment contents of the YS mutant, the sibling green seedlings (GS), and the two parental lines (Hwaseong and CR5029) and total carotenoid content of YS was significantly lower than that of GS and two parents. Using QTL-seq mapping, we identified *OsCHLI* as a candidate gene underlying the YS phenotype. A 2-bp deletion was detected in third exon, which is predicted to produce a truncated OsCHLI protein of 306 amino acids (AA). This deletion was not observed in the two parental lines, consistent with a spontaneous mutation. To further characterize the YS mutant, RNA-seq analysis was conducted. The expression of chlorophyll- and carotenoid-related genes were decreased in YS, and photosynthesis and carbohydrate gene pathway were significantly changed. Our study demonstrates that *OsCHLI* regulates leaf pigments, photosynthesis, and leaf structure development.

## 2. Results

### 2.1. Identification of YS Mutant

**Figure 1:**
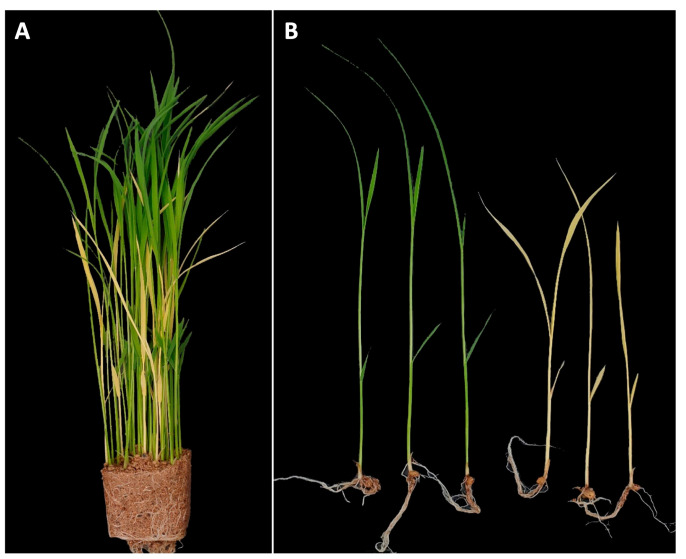
Identification of yellow seedling (YS) mutant. (**A**) Seedlings of CR5055-21 segregating for yellow seedlings and green seedlings. (**B**) Comparison of seedling morphology between normal green seedlings and yellow seedlings.

YS mutant phenotype was initially identified in segregating progeny of CR5055-21, a member of an F_2_ population (n = 440) from a cross between CR5029 and Hwaseong (Figure 1A). Chlorophyll, an essential pigment for photosynthesis, was absent in YS plants (Figure 1B), which were also slower to emerge and shorter than their GS siblings. In the YS plants, leaf necrosis and wilting were observed about two weeks after germination and eventually the YS plants died two to three weeks after germination. The observation that YS plants were only found in the progeny of the CR5055-21 line suggested that this phenotype was the result of (1) hybrid weakness or breakdown caused by a combination of more than two loci from the parental lines CR5029 and Hwaseong, which are frequently observed in the progeny of interspecific cross, or (2) a spontaneous mutation in CR5055-21. F_4_ seeds were harvested from 22 of the GS F_3_ plants that were transplanted in the field. Of these lines, 15 showed segregation for the YS trait and 7 showed only the GS phenotype. One of the segregating F_3_ lines, CR2030-1, produced abundant seeds which were used for characterization of the YS and GS phenotypes. To determine mode of inheritance for the YS phenotype, the segregation of leaf color in the CR2030-1 progeny (F_4_ seedlings) was investigated. Among the 437 seedlings, 339 and 98 of GS and YS were observed, respectively. The chi-square test for goodness of fit to a 3:1 segregation ratio (*χ*^2^ value = 1.545, df = 1, and *p* = 0.21) supports a single recessive gene mode of inheritance for the YS phenotype, which is consistent with a spontaneous mutation event.

### 2.2. Examination of Pigments in YS Mutant Leaves

**Figure 2:**
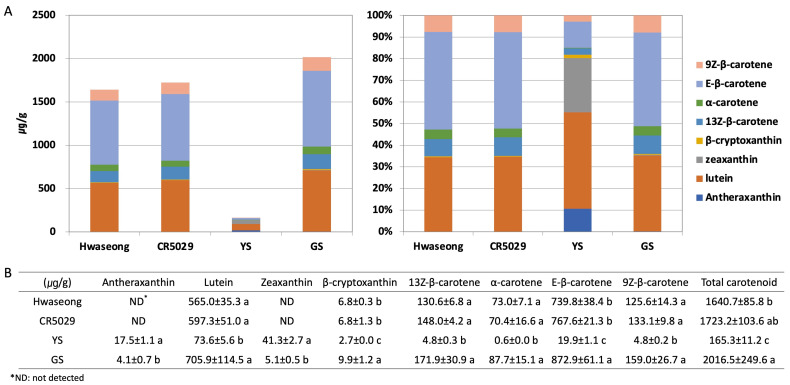
HPLC analysis of carotenoid content. Carotenoid content shown with (**A**) bar graph and (**B**) table. Data are represented by the mean ± standard deviation (n = 3). Letters indicate a significant difference at *p* < 0.05 based on Tukey’s test.

The chlorophyll a and b contents of Hwaseong, CR5029, and GS were very similar, however, those of YS were significantly lower than those of normal green plants (Supplementary Figure S1). Although YS have significantly reduced chlorophyll content, yellow pigments (i.e., carotenoids) are clearly present in the leaves unlike albino mutants (Supplementary Figure S2). HPLC analysis was performed to characterize the carotenoid content in the leaves of YS, GS, CR5029 and Hwaseong seedlings (Figure 2, Supplementary Figure S3). The total carotenoid content of YS was lower than CR5029, Hwaseong, and GS. In contrast, GS showed a higher total carotenoid content than YS and both parental lines (Figure 2A). E-β-carotene was the major species of carotenoids in the normal green plants (Hwaseong, CR5029, and GS), accounting for 43–45% of total carotenoid content. Lutein was the next most abundant carotenoid, comprising 34–45% of total carotenoid content, while the other carotenoids contributed less than 10% of the total content. In contrast, the major carotenoid in the YS mutant was lutein, accounting for 41% of total carotenoids, and zeaxanthin was the second highest component, with 25% of total carotenoids. Compared to normal green plants, the content of lutein, β-cryptoxanthin, 13Z-β-carotene, α-carotene, E-β-carotene, and 9Z-β-carotene was significantly decreased in YS plants (*p* < 0.05). Antheraxanthin and zeaxanthin were detected in YS and GS, whereas these two pigments were not found in Hwaseong and CR5029 (Figure 2B). In addition, the levels of antheraxanthin and zeaxanthin in the YS were significantly higher than in the GS (*p* < 0.05). Of the normal green plant lines, the two parents did not exhibit any significant differences in carotenoid content, however, GS plants displayed significantly higher β-cryptoxanthin and E-β-carotene contents than the parents and YS plants (Figure 2B). These results indicate that YS phenotype reflects both a deficiency in chlorophylls and an altered total carotenoid profile compared to the parents and GS.

### 2.3. Microscopy Revealed Differences in Leaf Anatomy and an Absence of Chloroplasts

**Figure 3:**
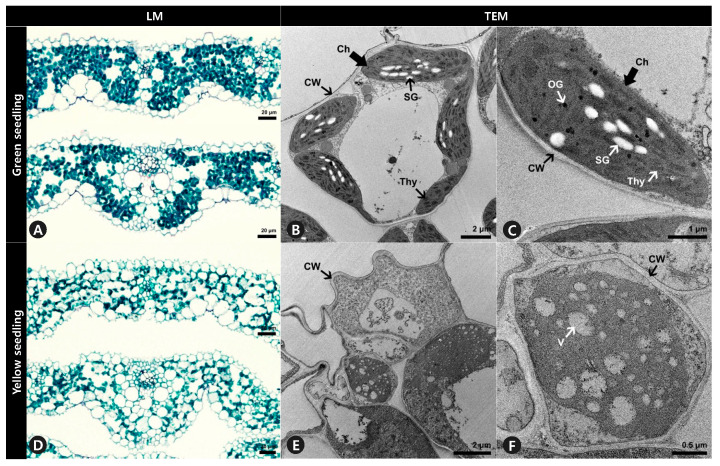
Leaf cross-section and ultrastructure of mesophyll cells of (**A**–**C**) green seedlings (GS) and (**D**–**F**) yellow seedlings (YS). (**A**,**D**) Leaf blades were double stained with Fast-Green FCF and Safranin O solutions. (**B**,**C**,**E**,**F**) Ultrastructure of mesophyll cells observation to investigate chloroplast of GS and YS. LM, light microscopy. TEM, transmission electron microscopy. *Ch*, chloroplast. *CW*, cell wall. *OG*, osmiophilic plastoglobuli. *SG*, starch granule. *Thy*, thylakoids. *V*, vacuole.

Anatomical analysis using light microscopy showed that the internal leaf structure differed significantly between the GS and YS. The mesophyll of the GS was compact, and the stained parenchyma was composed of numerous cells throughout the leaves (Figure 3A). However, the mesophyll of the YS was more loosely organized, and the stained parenchyma was sparse (Figure 3D). Ultrastructural analysis showed that the structure of chloroplasts from GS significantly differed from YS. The chloroplasts of GS leaves were normal in shape and contained thylakoid membranes with dense and well-structured grana stacks, starch granules, and osmiophilic plastoglobuli (Figure 3B,C). In contrast, the chloroplasts in YS leaves did not display the usual structure and had no observable grana lamella stacks (Figure 3E,F).

### 2.4. QTL-Seq Analysis Identified a Candidate Gene for the YS Phenotype

**Figure 4:**
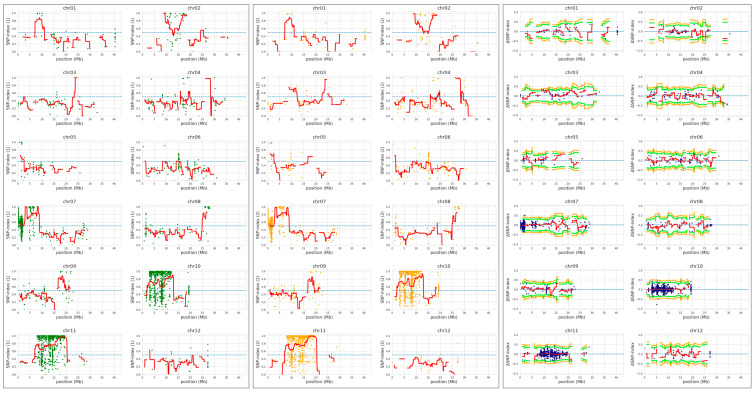
QTL-seq analysis to identify locus associated with the yellow seedling phenotype. Hwaseong was used for parental reference sequence in QTL-seq analysis. Single nucleotide polymorphism (SNP)-index plot of GS bulk (green) and YS bulk (orange), and delta-SNP index (blue) were shown. Red line indicates mean of SNP-indices, and orange and green line indicate mean of 99% and 95% confidence interval of simulated delta SNP-indices (p99 and p95), respectively.

QTL-seq was conducted to identify the YS locus, however, no significant QTL were detected from the QTL-seq sliding window analysis (Figure 4 and Supplementary Figure S4). CR5029, which harbors a small amount of *O. grandiglumis* segments in the Hwaseong genetic background, has low polymorphism compared with Hwaseong (Figure 4), and low polymorphism might lead to failure of QTL detection in the sliding window analysis. Since the YS trait exhibited a single recessive gene mode of inheritance, significant SNPs and InDels detected by QTL-seq at *p* < 0.01 were examined (Table 1). Fifteen sequence variations were identified, and their physical locations were determined. Among these variants, seven InDels were detected on the genic region and two InDels at Chr3: 20,248,858 and Chr9: 12,721,564 led frameshift mutation. Based on the gene description, we selected *OsCHLI* (*OsELL*) gene as a candidate of YS mutants, which harbors a 2-bp deletion at Chr3: 20,248,858. *OsCHLI* encodes one subunit of magnesium chelatase, which catalyzes the first committed step in chlorophyll biosynthesis. A point mutation in *OsCHLI* was previously reported to result in etiolated leaves and a lethal, chlorophyll-deficient (*OsELL*) seedling phenotype in rice [19]. For the 2-bp deletion, the delta-SNP index of YS and GS were 1 and 0.2157, respectively, indicating that all sequencing reads generated from the YS bulk group had the 2-bp deletion at Chr3: 20,248,858. Other 15 sequence variants were not located on the genic region or functionally unrelated genes with chlorophyll or carotenoid metabolism pathway (Table 1). These results indicate that the YS phenotype is strongly associated with the 2-bp deletion, making *OsCHLI* the likely gene underlying the YS phenotype in CR5055-21.

### 2.5. The 2-bp Deletion in OsCHLI Co-Segregated with YS

**Figure 5:**
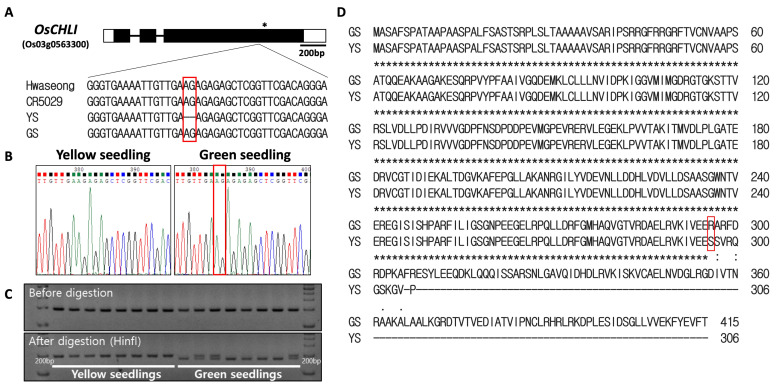
Sequence analysis of *OsCHLI*. (**A**) Gene structure and sequence comparison of Hwaseong, CR5029, yellow seedling (YS), and green seedling (GS). * indicates stop codon location. (**B**) Sanger sequencing confirmation of 2-bp AG deletion. (**C**) Genotyping of yellow and green seedlings with ELL_dCAPs marker. Green seedlings heterozygous at the locus show both bands after HinfI restriction digest. Full-length gel is presented in Supplementary Figure S5. (**D**) Alignment of predicted protein sequence from GS and YS alleles. Red box indicates corresponding position of the 2-bp nucleotide deletion.

Sanger sequencing of the *OsCHLI* gene was performed to confirm the presence of the 2-bp deletion in the YS mutant (Figure 5A). The deletion (AG) was detected in the third exon of the gene and was found in the YS plants, but was not in Hwaseong, CR5029, or GS (Figure 5A,B). The 2-bp deletion (AG) was found in a short dinucleotide repeat region (AG_4_) (Figure 5A,B). Based on these results, it is likely that the 2-bp deletion spontaneously occurred in CR5055-21 resulting in the appearance of the recessive YS phenotype in the progeny of this line. A dCAPs marker (ELL_dCAPs) was developed based on the deletion and confirmed to clearly distinguish YS and GS plants (Figure 5C, Supplementary Figure S5). The 2-bp deletion was predicted to lead to a frameshift resulting in a truncated protein in YS plants (Figure 5D). Upon screening a population (n = 275) of segregating F_4_ plants, the YS phenotype was found to completely co-segregate with the ELL_dCAPs marker genotype.

### 2.6. Prediction and Alignment of the Protein Structure of OsCHLI

**Figure 6:**
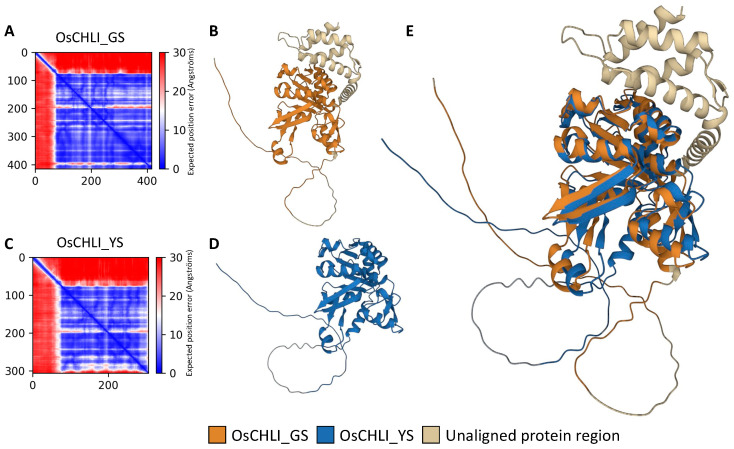
Protein structure prediction and pairwise structure alignment of OsCHLI. (**A**,**C**) Visualization of predicted alignment error of OsCHLI_GS and OsCHLI_YS, (**B**,**D**) Predicted protein structure of OsCHLI_GS and OsCHLI_YS generated by ColabFold. (**E**) Alignment of two protein structures.

To examine the effect of the 2-bp deletion on the OsCHLI protein, ColabFold was employed to predict and compare the structures OsCHLI from GS (OsCHLI_GS) and YS (OsCHLI_YS). Prediction results revealed that the alignment error of the N-terminus region of OsCHLI_GS and OsCHLI_YS was very high, indicating that the prediction accuracy of this region was low (Figure 6A,C). However, the other amino acid sequences of OsCHLI_GS and OsCHLI_YS were predicted with high accuracy, and the 3-dimensional protein structures of OsCHLI_GS and OsCHLI_YS were successfully predicted (Figure 6B,D). Two predicted structures were aligned with pairwise protein alignment, and the two structures overlapped with high similarity (Figure 6E). The unaligned protein is shown in beige and corresponds to the truncated region of OsCHLI_YS (Figure 6E). 

### 2.7. The Mutation in OsCHLI Altered the Expression of Photosynthesis and Carbohydrate Metabolism Pathway Genes

**Figure 7:**
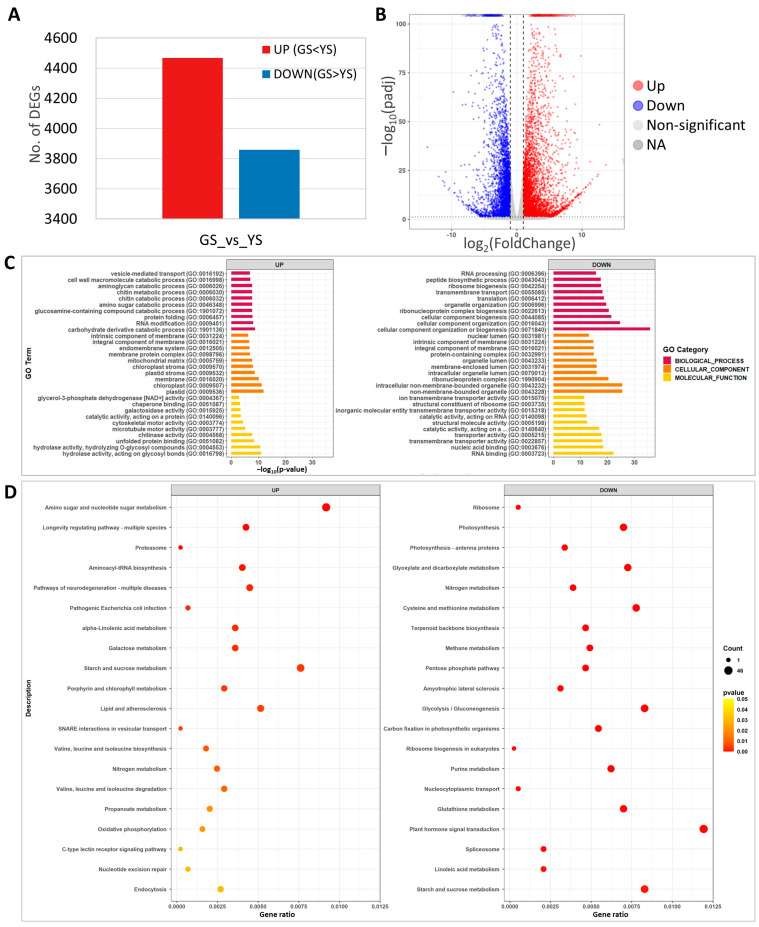
Transcriptome analysis of green seedlings (GS) and yellow seedlings (YS). (**A**) Number of differentially expressed genes (DEGs) from comparison of yellow and green seedling transcriptomes. (**B**) Volcano plot of significantly up- or down-regulated DEGs. (**C**) Gene ontology enrichment analysis of the DEGs. (**D**) KEGG pathway enrichment analysis of DEGs.

RNA-seq analysis was carried out using leaf samples from 10-day-old YS and GS seedlings. A total of 8327 DEGs were identified between the YS and GS. Among the 8327 DEGs, 4468 and 3859 genes were up-regulated and down-regulated, respectively (Figure 7A, Supplementary Tables S1 and S2). The volcano plot showed distribution of a statistical significance and altered expression level of the DEGs between YS and GS (Figure 7B).

GO analysis of the 8327 DEGs identified carbohydrate derivative catabolic process (37 DEGs) for biological processes, plastid (279 DEGs) for cellular components, and hydrolase activity acting on glycosyl bonds (128 DEGs) for molecular function as the most significant up-regulated DEGs (Figure 7C). Among the down-regulated DEGs, cellular component organization or biogenesis (133 DEGs) for biological processes, non-membrane-bounded organelles (88 DEGs) for cellular components, and RNA binding (50 DEGs) for molecular function were the most significant (Figure 7C). In the significantly enriched GO, terms of up-regulated DEGs, plastid, chloroplast, plastid stroma, chloroplast stroma, and carbohydrate derivative catabolic process that are associated with photosynthesis and chloroplast development were identified, likely the result of the abnormal function of magnesium chelatase in YS.

For KEGG pathway analysis, the top 20 significant categories were identified for up- and down-regulated DEGs. The up-regulated DEGs included amino sugar and nucleotide sugar metabolism, starch and sucrose metabolism, and porphyrin and chlorophyll metabolism (Figure 7D). KEGG analysis identified photosynthesis, terpenoid backbone biosynthesis, plant hormone signal transduction, and starch and sucrose metabolism in the down-regulated DEGs (Figure 7D). These results indicated that YS showed significant changes in transcriptome expression and that the loss of function of *OsCHLI* affected the expression of photosynthesis, starch and sugar biosynthesis, and pigment-related genes.

### 2.8. Leaf Pigment- and Photosynthesis-Related Gene Expression Was Changed in YS Mutant

**Figure 8:**
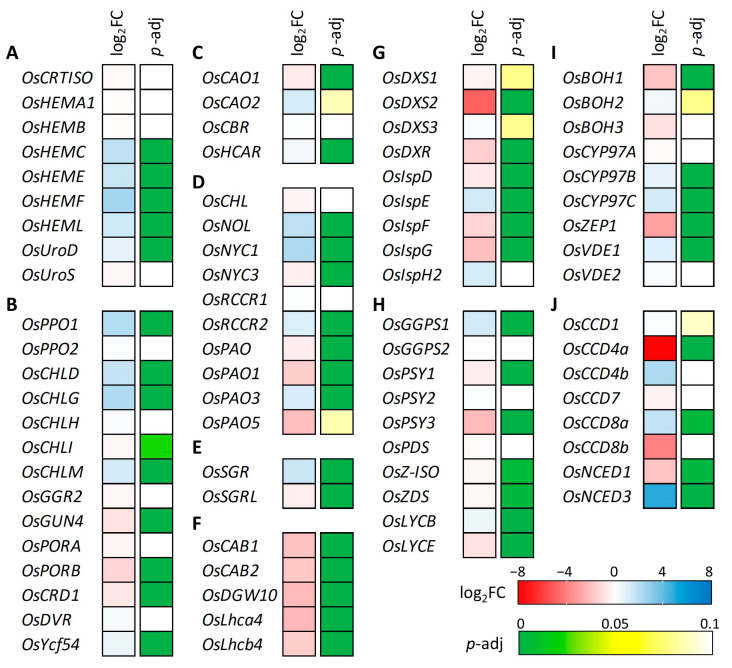
Gene expression analysis of chlorophyll and carotenoid-associated genes. (**A**) Common pathway, (**B**) Chlorophyll-specific biosynthesis pathway, (**C**) Chlorophyll cycle pathway, (**D**) Chlorophyll degradation pathway, (**E**) LHC degradation pathway, (**F**) LHC synthesis pathway, (**G**) Carotenoid substrate supply (MEP pathway), (**H**) Carotene biosynthesis pathway, (**I**) Xanthophyll biosynthesis pathway, and (**J**) Carotenoid degradation pathway. Different gene expression was determined by DEseq2 and red and blue color indicate decreased and increased gene expression level in yellow seedling compared to green seedling. Adjusted *p* value was used for statistically significance level of difference based on the Wald test.

To further characterize the effect of the *OsCHLI* YS allele, the expression levels of photosynthesis- and leaf pigment-related (chlorophylls and carotenoids) genes were investigated from the transcriptome data (Figure 8, Supplementary Table S3). Common pathway genes of chlorophyll synthesis (*OsHEMC, OsHEME*, *OsHEMF*, and *OsHEML*) showed increased expression in the YS (Figure 8A). The expression levels of the three subunits of Mg-chelatase were examined, and only *OsCHLD* was up-regulated in YS based on the DEG classification (Figure 8B). While the YS allele was predicted to yield a truncated protein, transcript levels of *OsCHLI* were not significantly different between YS and GS. In the LHC degradation pathway genes, *OsSGR* showed up-regulated transcript level in YS while the expression level of *OsSGRL* was decreased in YS (Figure 8E). The expression of light-harvesting complex (LHC) synthesis pathway genes (*OsCAB1*, *OsCAB2*, *OsDGW10*, *OsLhca4*, and *OsLhcb4*) was down-regulated in YS (Figure 8F). Some chlorophyll degradation pathway genes, also known as leaf senescence-related genes, were up-regulated including *OsNOL*, *OsNYC1*, *OsRCCR2*, and *OsSGR*. Many genes of the carotenoid substrate supply and biosynthesis pathway were down-regulated in YS (Figure 8G,H). Among these carotenoid-related genes, *CCD4a* encoding carotenoid cleavage dioxygenase showed the most decreased expression in YS. Although many carotenoid-related genes were down-regulated, *NCED3* encoding 9-cis-epoxycarotenoid dioxygenase was significantly up-regulated with a four to five-fold increase in YS (Figure 8J). Our findings show that the expression of a wide array of photosynthesis-, chlorophyll-, and carotenoid-related genes were altered in the YS mutant. 

## 3. Discussion

In this study, we identified a lethal, chlorophyll-deficient, yellow seedling phenotype in the line CR5055-21, which was derived from an interspecific backcross between Hwaseong and the wild species *O. grandiglumis*. QTL-seq and sequencing analyses revealed that the sequence variation was not derived from either parent, thus indicating that a spontaneous mutation likely occurred in the CR5055-21 F_2_ plant, resulting in the appearance of segregating YS and GS plants in the F_3_ (Figure 1A). Analysis of F_4_ progeny from GS lines confirmed a single recessive gene mode of inheritance of the YS phenotype and a 2-bp deletion was identified in *OsCHLI*, which encodes one of the three subunits of Mg-chelatase. This critical enzyme catalyzes the first committed step of chlorophyll biosynthesis [19].

The 2-bp deletion (AG) was detected in a short dinucleotide repeat region (AG_4_) (Figure 5). In the F_2_ population derived from a backcross between introgression line CR5029 and Hwaseong, we identified another leaf color mutant line which has 1-bp T deletion in a repeated T sequence region (unpublished data). As with the 2-bp deletion in YS, the 1-bp T deletion was not derived from either parent. Thus, two leaf color mutants were found in the same cross population and were likely the result of spontaneous mutations in repeat sequence regions. This population could have high frequency of DNA slippage mutations. To clarify this, we are screening for other spontaneous mutations on a genome-wide scale using the bulked DNA sequences of YS and GS and further analysis will be conducted.

A *CHLI* mutant of strawberry generated by N-Ethyl-N-nitrosourea (ENU) displayed yellow-green leaves with decreased carotenoid content compared to its wildtype [26]. In addition, rice *ygl7* (*yellow-green leaf 7*) mutant which has non-synonymous SNP in *OsCHLD* showed significantly reduced carotenoids content [27]. These studies indicated that magnesium chelatase subunits may be associated with regulation of carotenoid content. However, these two studies measured only total carotenoid content using spectrophotometer and changes of carotenoids were not specifically examined. In addition, most of the carotenoid biosynthesis studies in rice have focused on the accumulation of carotenoids in rice endosperm to develop golden rice [28]. In our carotenoid profiling of YS, GS, and the parental lines, significantly altered carotenoid content was observed and specific carotenoids were identified by HPLC analysis (Figure 2). While the results of the pigment content analysis of YS was consistent with the phenotype (i.e., yellow leaves, seedling lethal), somewhat surprisingly, GS showed a significantly higher total carotenoid content, and the β-cryptoxanthin and E-β-carotene species were significantly higher than the two parental lines. It is unclear whether the mutation in *OsCHLI* caused the changes in zeaxanthin and antheraxanthin content in the YS and GS. It should be noted that the zygosity of the *OsCHLI* gene in the GS samples used for pigment analysis was not determined and could have included both homozygous and heterozygous individuals (i.e., *OsCHLI^GS^*/*OsCHLI^GS^* or *OsCHLI^GS^*/*OsCHLI^YS^*). To determine if the 2-bp deletion in *OsCHLI* contributes to altered carotenoid profile, the carotenoid contents of homozygous and heterozygous GS samples need to be examined. 

The 2-bp deletion in *OsCHLI* may be indirectly responsible for the altered carotenoid profiles of YS and GS plants by affecting the expression of genes involved in carotenoid metabolism genes. Among the genes identified by RNA-seq analysis, *carotenoid epsilon-ring hydroxylase* (*OsCYP97C*) was up-regulated in the YS, whereas *phytoene synthase 3* (*OsPSY3*), *beta-carotene hydroxylase* (*OsBOH1*), and *zeaxanthin epoxidase* (*OsZEP1*) were down-regulated. *OsZEP1* plays a crucial role as an enzyme responsible for transforming zeaxanthin into antheraxanthin and violaxanthin [29]. In YS plants, the expression of *OsZEP1* was significantly reduced by approximately threefold. This decrease in expression likely led to higher levels of zeaxanthin and antheraxanthin in YS plants (Figure 2 and Figure 8I). The reduced expression of *OsZEP1* could potentially disrupt the conversion of zeaxanthin to the next step, resulting in an accumulation of zeaxanthin and antheraxanthin. In the carotenoid degradation pathway, the expression levels of *OsCCD4a* and *OsNCED1* were decreased (Figure 8J). *OsCCD4a*, encoding carotenoid cleavage dioxygenase, plays an important role in regulating carotenoid degradation. It has been reported that suppressed expression of *OsCCD4a* enhances leaf carotenoids in rice RNAi lines by reducing carotenoid degradation [28]. Leaf carotenoids in YS were lower than in GS, and transcript levels of *OsCCD4a* in YS decreased 7–8 fold compared to GS, suggesting a feedback control mechanism of gene expression by the low carotenoid production in YS. In addition, decreased expression level of MEP, carotenoid biosynthesis, and xanthophyll biosynthesis genes may be associated with decreased carotenoid content in YS (Figure 8G–I). Altered expression of carotenoid-related genes supports decreased carotenoid content in YS mutant.

Transcriptome analysis using green-revertible albino leaf and wildtype in rice revealed that DEGs were enriched in the terms of thylakoid, chloroplast, photosynthetic membrane at the cellular component category, and in the terms of carbohydrate derivative binding and tetrapyrrole binding at molecular function category [30]. In addition, most DEGs were annotated in the metabolic pathway. In our study, RNA-seq analysis showed that DEGs related with chloroplast, photosynthesis, carbohydrate, and sugar metabolic pathway were enriched in GO and KEGG analysis (Figure 7C,D). These enrichment patterns of DEGs are consistent with the chlorophyll and photosynthesis deficiency in YS. 

Ultrastructural observations revealed that chloroplasts were not observed in YS seedlings, suggesting that the truncated OsCHLI protein prevented chloroplast development in this mutant (Figure 3E,F). The chlorophyll-deficient *ell* mutant, which has a point mutation in *OsCHLI,* similarly exhibited undeveloped chloroplast and thylakoid structures [19]. These results are consistent with the role of *OsCHLI* in chlorophyll biosynthesis and chloroplast development. The T-DNA insertion mutant of *OsCHLH* has a lethal yellow seedling phenotype like that of *OsCHLI* mutants (YS and *ell*), but abnormal chloroplasts with disrupted thylakoid membranes were observed [15]. This may suggest different functions relating to chloroplast development for the *OsCHLI* and *OsCHLH* subunits of Mg-chelatase. In addition to the lack of chloroplasts, the mesophyll structure of YS was looser than that of GS, and fewer parenchyma were present in YS (Figure 3). Changes in leaf anatomy and plant morphology have been observed in chlorophyll-deficient mutants of other species such as *D. regia* albino seedlings [31] and tree peony (*Paeonia suffruticosa*) [32]. These results indicated that chlorophyll-deficient phenotype and mutations in magnesium chelatase can affect the development of leaf tissues.

QTL-seq analysis did not successfully identify a candidate locus for the YS phenotype. Since the sliding window analysis uses the average value of the SNP and delta-SNP index with a 2-Mbp region, the failure to detect a significant locus was likely due to the lack of polymorphism between CR5029 and Hwaseong, the parents of the CR5055-21 line. CR5029 is an introgression line generated by crossing Hwaseong with the wild species *O. grandiglumis* followed by backcrossing several times with Hwaseong. As a result, only a few *O. grandiglumis* alleles were introgressed in CR5029. In fact, the 2-bp deletion in *OsCHLI* was located on chromosome 3, which had less than 100 sequence variations (SNP and InDel) between Hwaseong and CR5029. Thus, QTL-seq analysis may not be effective for genetically close populations such as introgression lines and backcrossed populations. 

The deletion in the *OsCHLI_YS* allele is predicted to lead to a frameshift and the generation of a truncated protein of 306 AA compared to the normal protein of 415 AA. OsCHLI has a putative chloroplast transit peptide (AA 1-67) and an AAA+ ATPase domain (AA 93-415) [17,33]. The AAA+ ATPase proteins are involved in the processes of protein folding and unfolding, assembly or disassembly of protein complexes, protein transport, and protein degradation [34]. A point mutation which led amino acid change (G177R) in the AAA+ ATPase domain of *OsCHLI* blocks the interaction between OsCHLI and OsCHLD and induces etiolated leaves and a lethal (*ell*) phenotype [19]. Given the predicted truncation encoded by the YS allele, it is likely that the Mg-chelatase complex is not formed in the mutant seedlings and that the resulting loss of function is responsible for the chlorophyll-deficient phenotype. The *ell* allele of the *OsCHLI* exhibited semidominant gene action, showing light-green leaf phenotype in heterozygous *ELL*/*ell* plants [19]. However, GS individuals of homozygous and heterozygous *OsCHLI* (*OsCHLI^GS^*/*OsCHLI^GS^* and *OsCHLI^GS^*/*OsCHLI^YS^*) were not visibly distinguished (Figure 1A). One possible explanation for this apparent difference in the effect of the *ell* and YS alleles in heterozygotes may be differences in the ability to interact with the other subunits of Mg-chelatase. In the heterozygous plant, OsCHLI^ell^ interrupted the binding of wildtype OsCHLI to form magnesium chelatase complex, resulting in decreased enzymatic activity of magnesium chelatase. Further study is required to determine if OsCHLI^YS^ can bind to the other subunits.

## 4. Materials and Methods

### 4.1. Plant Materials

The introgression line CR5029, which is derived from an interspecific cross between Hwaseong (*O. sativa*) and *O. grandiglumis*, was backcrossed with Hwaseong [35]. Recurrent parent Hwaseong was developed via another culture method of F_1_ plant derived from a cross between Aichi 37 and Samnambyeo [35]. *O. grandiglumis* and Hwaseong were originally obtained from the International Rice Research Institute (IRRI) and Rural Development Administration (RDA) in Korea, respectively. The procedure of developing interspecific hybrids and advanced backcross population between Hwaseong and *O. grandiglumis* was described in previous studies [36,37]. In the F_3_ population, the CR5055-21 line showed segregation for YS and GS (Figure 1A). The CR5055-21 F_3_ seedlings exhibiting normal green color (i.e., the GS individuals) and the parental lines CR5029 and Hwaseong were transplanted in the experimental paddy field at Chungnam National University (Daejeon, Republic of Korea) in mid-May, about one month after sowing in the greenhouse. Among these transplanted F_3_ lines, CR2030-1 which has abundant seeds showed segregation of GS and YS and was used for further experiments.

### 4.2. Anatomical and Ultrastructural Observation

To investigate transverse sections of GS and YS leaves, 10-day-old leaf samples were dehydrated in a tertiary butyl alcohol series (TBA) and processed for paraffin embedding using an automatic tissue processor (Leica EG1150H; Leica Microsystems, Wetzlar, Germany). The tissue blocks were sectioned using a manual rotary microtome (HistoCore MULTICUT; Leica Biosystems, Nussloch, Germany), and 5–10 μm sections were placed onto glass slides. Using an automatic slide stainer, the sections were double-stained in Fast-Green FCF and Safranin O solutions. The permanent slides were examined using an Olympus BX-53 light microscope (Olympus, Tokyo, Japan), and images were captured using an Olympus DP21 digital camera (Olympus, Tokyo, Japan).

For transmission electron microscopy (TEM), the dehydrated GS and YS leaves were collected to rehydrate in 0.05 M sodium cacodylate buffer (pH 7.2) for washing at room temperature for 5 min. All incubations were carried out in a microwave (PELCO BioWave^®^ Pro+; Ted Pella Inc., Redding, CA, USA) with the temperature limit set to 35 °C. The materials were post-fixed in 1% osmium tetroxide for 40 s in the microwave and washed at room temperature for 5 min. Following dehydration in a graded ethanol series, the transition of the dehydrated material was performed at room temperature for 40 s in a microwave oven. For infiltration, the samples were placed in a mixed solution of Spurr’s resin (Agar Scientific, Essex, UK) for 15 min in a microwave, followed by polymerization at 70 °C for 24 h. Sections were cut with an MT-X ultramicrotome (RMC; Boeckeler Instruments, Tucson, AZ, USA) and stained with 2% uranyl acetate for 7 min at room temperature, then with Reynolds lead citrate for 7 min. The sections were placed on copper grids, examined using TEM (LIBRA-120 energy-filtering TEM; Carl Zeiss, Oberkochen, Germany), and photographed.

### 4.3. Measurement of Chlorophylls and Carotenoids

Chlorophyll content of YS, GS, Hwaseong, and CR5029 was evaluated as described by [38]. The leaf blade of 10-day-old seedlings of parental lines, YS, and GS were bulked from 10 plants, and YS and GS were collected from segregating F_4_ seedlings. Total chlorophyll was extracted from 20 mg of fresh leaf sample using ice-cold 80% acetone. Supernatants were transferred to a cuvette, and the absorbance at 663 and 645 nm was measured using a UV/VIS spectrophotometer (Hanson Tech., Seoul, Republic of Korea).

Carotenoid content was evaluated using HPLC analysis according to previously reported methods [39,40]. Carotenoids were extracted from freeze-dried leaf blade samples of GS, YS, Hwaseong, and CR5029 10-day-old seedlings. The samples were ground using a TissueLyser II (QIAGEN, CA, USA) with 5 mm stainless steel beads. The YS (50 mg), GS (30 mg), Hwaseong (30 mg), and CR5029 (30 mg) samples were mixed with 3 mL of 0.1% ascorbic acid in ethanol (*w*/*v*) in 15 mL tubes, and then incubated in a water bath at 85 °C for 5 min. The samples were saponified by adding 120 μL of 80% potassium hydroxide in water (*w*/*v*) at 85 °C for 10 min and rapidly cooled on the ice for 5 min to stop the reaction. Next, 100 μL of 25 ppm trans-β-Apo-8′-carotenal (internal standard), water (1.5 mL), and hexane (1.5 mL) were added to the samples, which were vortexed and centrifuged for 5 min at 4 °C at 1200× *g*. After centrifugation, the supernatants (hexane layer) were transferred to new 15 mL tubes, and the extraction was repeated with hexane (1.5 mL). The isolated hexane layers (approximately 3 mL) were concentrated using nitrogen gas and a vacuum concentrator VS-802F (Visionbionex, Bucheon, Republic of Korea). Concentrates were dissolved in 250 μL of methanol: dichloromethane (1:1, *v*/*v*) solution and filtration was performed with a 0.5 μm syringe filter into a 2 mL autosampler vial. The carotenoids in the filtrates were separated on a YMC carotenoid HPLC column (250 × 4.6 mm, 3 μm i.d.; YMC Co., Kyoto, Japan) using an Agilent 1100 HPLC system (Agilent Technologies, Waldbroon, Germany) equipped with a diode array detector with the wavelength set at 450 nm. The column was set at 40 °C, and solvents A and B comprised methanol: water (92:8, *v*/*v*) with 10 mM ammonium acetate and 100% methyl tert-butyl ether, respectively. The binary gradient elution system of solvent A–solvent B was as follows: 0 min, 90% A/10% B; 20 min, 83% A/17% B; 29 min, 75% A/25% B; 35 min, 30% A/70% B; 40 min, 30% A/70% B; 42 min, 25% A/75% B; 45 min, 90% A/10% B; and 55 min, 90% A/10% B. Quantification of carotenoids was carried out by obtaining a calibration curve using standard compounds and calculating the ratio of the peak area of the standard compound to the peak area of the internal standard (Supplementary Figure S3).

### 4.4. DNA Extraction and PCR

Genomic DNA was isolated from fresh leaves using the CTAB method [41], and PCR was performed as described previously [42] with minor modifications. The PCR profile was: 95 °C for 5 min, followed by 40 cycles of 95 °C for 30 s, 55 °C for 30 s, and 72 °C for 30 s, and 5 min at 72 °C for the final extension. For genotyping of dCAPs markers (ELL_dCAPs; forward:5′-ATCCCTGTCGAACCGAGCTGACT-3′, reverse:5′-ATGGAACACCGTGGAGAGAG-3′), PCR amplicons were digested by HinfI restriction enzyme according to the manufacturer’s instructions (NEB, Ipswich, MA, USA). PCR products were mixed with loading dye, separated on 3% metaphor agar (TaKaRa, Otsu, Japan), and stained with StaySafe Nucleic Acid Gel Stain (RBC, New Taipei City, Taiwan). Sequencing of the PCR product was performed using the Solgent sequencing service (SolGent Co., Ltd., Daejeon, Republic of Korea). 

### 4.5. QTL-Seq Analysis

For the QTL-seq analysis, two DNA bulks (GSB and YSB) were pooled based on the phenotype. A total of 29 GS and 20 YS plants were included in GSB and YSB, respectively. Sequencing libraries of the two bulks were prepared using the TruSeq DNA Nano Kit (Illumina Inc., San Diego, CA, USA). Paired-end sequencing was conducted on the Illumina NovaSeq platform (Illumina Inc., San Diego, CA, USA). QTL-seq analysis was performed using the QTL-seq pipeline (ver. 2.1.3) [43,44]. Raw sequencing reads were trimmed using the Trimmomatic software in the QTL-seq pipeline [45]. Trimmed reads were mapped to the rice reference genome IRGSP-1.0 (https://rapdb.dna.affrc.go.jp/download/irgsp1.html; accessed on 23 June 2023), and variants were identified. SNP and delta-SNP indices were calculated, and the sliding window approach was employed for the average SNP and delta-SNP indices with a 2-Mb window size and 100-kb increment. Based on the average SNP and delta-SNP indices, dot plots and trend lines were generated for the 12 chromosomes. QTL were defined when the average delta-SNP index was significantly greater than the surrounding region and exhibited an average *p* < 0.05.

### 4.6. Protein Structure Prediction and Alignment

Protein structure prediction analyses were conducted using ColabFold: AlphaFold2 using MMseqs2 (https://colab.research.google.com/github/sokrypton/ColabFold/blob/main/AlphaFold2.ipynb#scrollTo=kOblAo-xetgx; accessed on 23 June 2023) in pdb70 template mode [46]. The amino acid sequences of OsCHLI wild-type (OsCHLI_GS) and yellow seedling (OsCHLI_YS) were input, and five putative structures were predicted for each amino acid sequence. The five were ranked based on their structural accuracy. For pairwise structure alignment, the highest ranked structures of OsCHLI_GS and OsCHLI_YS were used, and alignment was conducted in the RCSB PDB Pairwise Structure Alignment menu in jFATCAT (rigid) mode (https://www.rcsb.org/alignment; accessed on 23 June 2023) [47,48,49].

### 4.7. RNA-Seq Analysis

Total RNA was isolated from bulks of 20 GS and 20 YS leaf samples at 10-day-old stage using RNAiso plus following the manufacturer’s instructions (TaKaRa, Otsu, Japan). RNA-seq libraries were constructed independently for three biological replicates of GS and YS samples using an Illumina TruSeq RNA Sample Preparation Kit, according to the manufacturer’s protocol (Illumina Inc., San Diego, CA, USA). Sequencing data were generated using an Illumina HiSeq X platform and the raw reads were filtered using Trimmomatic and BBduk to remove adapters, low-quality reads, and contaminated reads [45]. Read mapping was conducted using HISAT2 on the IRGSP-1.0 reference genome, and transcript abundances were determined using the htseq-count method [50,51]. DESeq2 normalized gene expression levels, and normalized data were used to identify differentially expressed genes (DEGs) when the expression level was different with more than 2-fold change at adjusted *p* < 0.05 [52]. Gene ontology (GO) enrichment and Kyoto Encyclopedia of Genes and Genomes (KEGG) pathway enrichment analyses were carried out for up- and down-regulated DEGs using the Blast2Go and KEGG Automatic Annotation Servers, respectively [53,54].

## 5. Conclusions

Previously, Mg-chelatase subunit genes *OsCHLD*, *OsCHLH*, and *OsCHLI* have been characterized. However, the regulatory mechanisms of these genes remain poorly understood. In our study, a novel mutant allele of *OsCHLI* is associated with a severe chlorophyll deficiency, disrupted chloroplast development, altered carotenoid content, and changes in expression of an array of leaf pigment metabolism and photosynthesis-related genes. These phenotypic changes are consistent with the important role that Mg-chelatase plays in chlorophyll biosynthesis and underscore its connection with chloroplast development, leaf anatomy, and the metabolism of other leaf pigments. Our findings contribute towards a better understanding of the complex mechanisms governing leaf pigment biosynthesis in rice.

## Figures and Tables

**Table 1:** Significant sequence variants detected from QTL-seq analysis at *p* < 0.01.

Chr.	Position	Variant	p99	SNP Index			Gene ID	Gene Name	Variant Location	Description
				GS	YS	Delta				
3	20,248,858	indel	0.41	0.22	1.00	0.78	Os03g0563300	*ELL*, *OsCHLI*	Exon (Frameshift)	Magnesium-chelatase subunit ChlI, Mg-chelatase I subunit
3	20,693,214	indel	0.53	0.12	0.76	0.65	Os03g0570300		Promoter	Zinc finger, C2H2-like domain-containing protein.
5	3,101,365	indel	0.48	0.00	1.00	1.00	Intergenic			
7	24,595,098	indel	0.48	1.00	0.00	−1.00	Os07g0602200		Intron	Similar to HDA1.
7	24,595,109	indel	0.49	0.05	1.00	0.95	Os07g0602200 Intergenic		Intron	Similar to HDA1.
7	24,599,596	indel	0.46	0.00	0.76	0.76				
7	26,805,909	indel	0.59	0.00	1.00	1.00	Intergenic			
9	12,636,359	indel	0.49	0.00	0.91	0.91	Intergenic			
9	12,721,564	indel	0.44	0.03	1.00	0.97	Os09g0378100		Exon (Frameshift)	ND *
9	12,832,179	indel	0.54	0.00	0.94	0.94	Os09g0380300		Exon	Hypothetical conserved gene
11	8,522,066	indel	0.52	0.05	1.00	0.95	Intergenic			
11	9,368,578	indel	0.49	1.00	0.00	−1.00	Intergenic			
12	5,109,930	indel	0.47	0.05	0.94	0.89	Intergenic			
12	5,702,114	indel	0.48	0.09	0.93	0.84	Os12g0209200	*OsBBX30*	Exon	Similar to CONSTANS-LIKE a.
12	6,749,621	indel	0.49	0.94	0.00	−0.94	Intergenic			

* ND: not determined.

## Data Availability

All the data generated or analyzed during this study are included in this published article and its supplementary information files. Sequence data for QTL-seq and RNA-seq were deposited in NCBI SRA (PRJNA935477).

## Supplementary Materials

The following supporting information can be downloaded at: https://www.mdpi.com/article/10.3390/plants12152831/s1, Supplementary Figure S1. Comparison of chlorophyll a and b content in the Hwaseong, CR5029, Green seedling, and yellow seedling. Letters on the bar graph indicate a significant difference at *p* < 0.05 based on Tukey’s test. Supplementary Figure S2. Comparison of normal green, yellow, and albino seedlings which is derived from a cross between CR5029 and Hwaseong population. Supplementary Figure S3. HPLC chromatogram of carotenoids obtained from leaves of (A) Hwaseong, (B) CR5029, (C) YS, and (D) GS. Peak: 1. Antheraxanthin; 2. Lutein; 3. Zeaxanthin; 4. trans-β-Apo-8′-carotenal (internal standard); 5. β-Cryptoxanthin; 6. 13Z-β-Carotene; 7. α-Carotene; 8. E-β-Carotene; 9. 9Z-β-Carotene. Supplementary Figure S4. QTL-seq analysis to identify locus associated with the yellow seedling phenotype. CR5029 was used for parental reference sequence in QTL-seq analysis. Single nucleotide polymorphism (SNP)-index plot of GS bulk (green) and YS bulk (orange), and delta-SNP index (blue) were shown. Red line indicates mean of SNP-indices, and orange and green line indicate mean of 99% and 95% confidence interval of simulated delta SNP-indices (p99 and p95), respectively. Supplementary Figure S5. PCR amplicons of ELL_CAPs marker before and after digestion with restriction enzyme HinfI. Amplicons were separated on 3% Agarose gel stained with StaySafe Nucleic Acid Gel Stain (RBC). Supplementary Table S1. List of up-regulated DEGs. Supplementary Table S2. List of down-regulated DEGs. Supplementary Table S3. List of chlorophyll and carotenoid-associated genes.
