# Supplementary material for: A Frameshift Mutation in the Mg-Chelatase I Subunit Gene OsCHLI Is Associated with a Lethal Chlorophyll-Deficient, Yellow Seedling Phenotype in Rice
Source: Plants (Basel). 2023 Jul 31;12(15):2831. doi: 10.3390/plants12152831 (PMC10420988; doi:10.3390/plants12152831)
Supplement: Supplementary file 1 [file plants-12-02831-s001.zip › YS_Supplementary_figures.pdf]

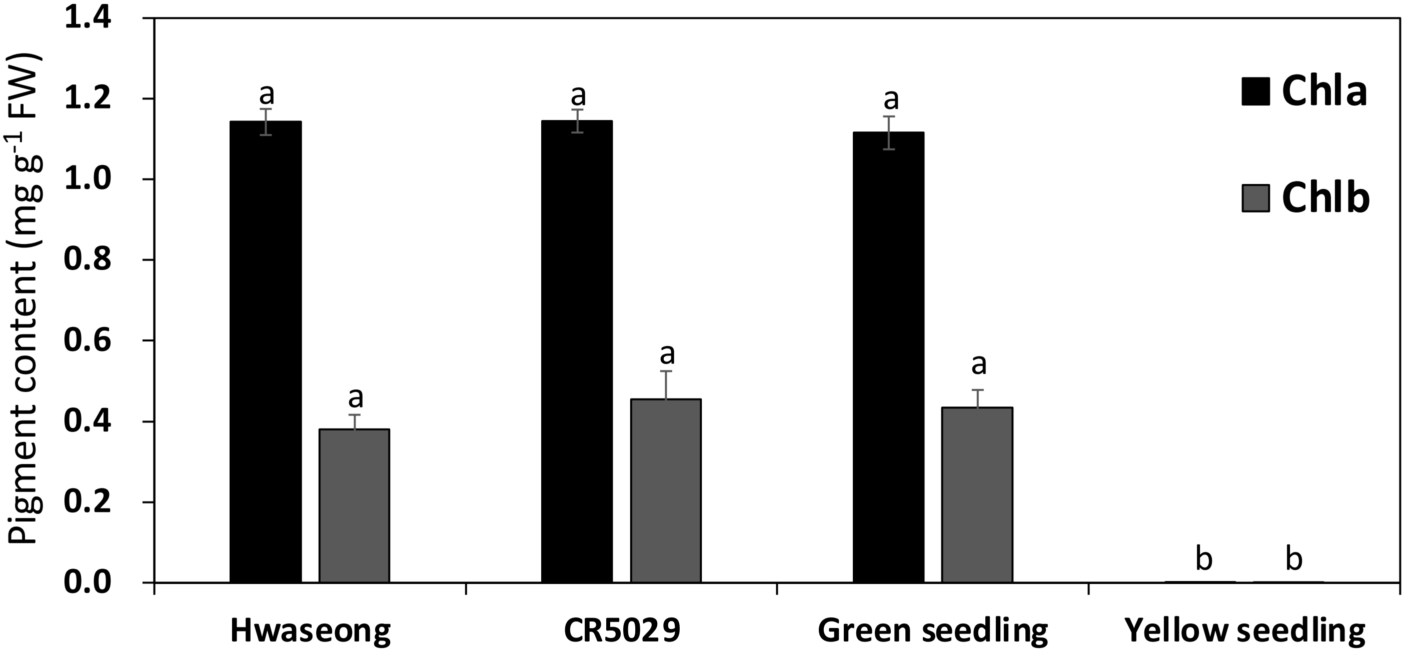

Figure S1. Comparison of chlorophyll a and b content in the Hwaseong, CR5029, green seedling, and yellow seedling. Letters on the bar graph indicate a significant difference at $p < 0.05$ based on Tukey's test.

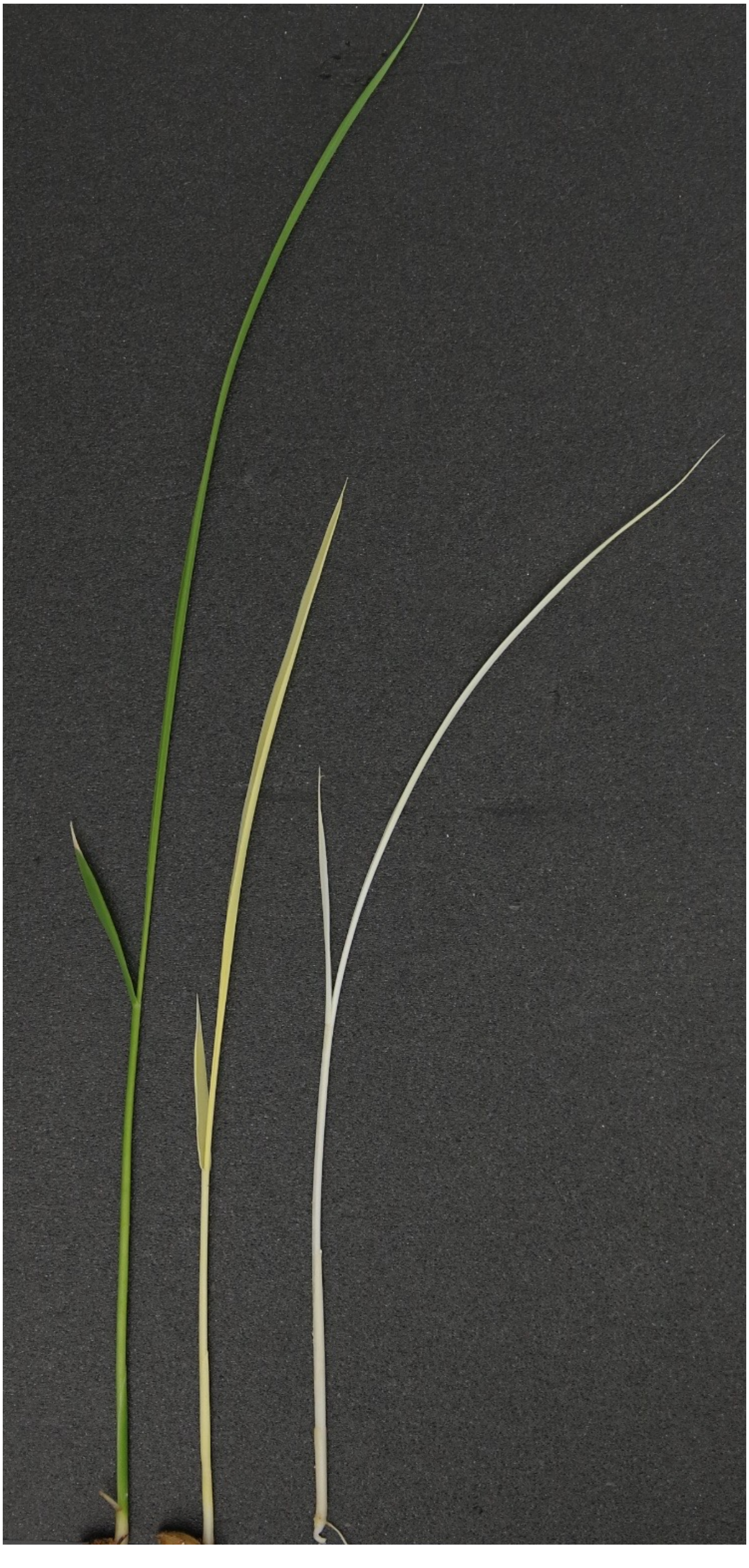

Figure S2. Comparison of normal green seedling, yellow seedling and albino seedling.

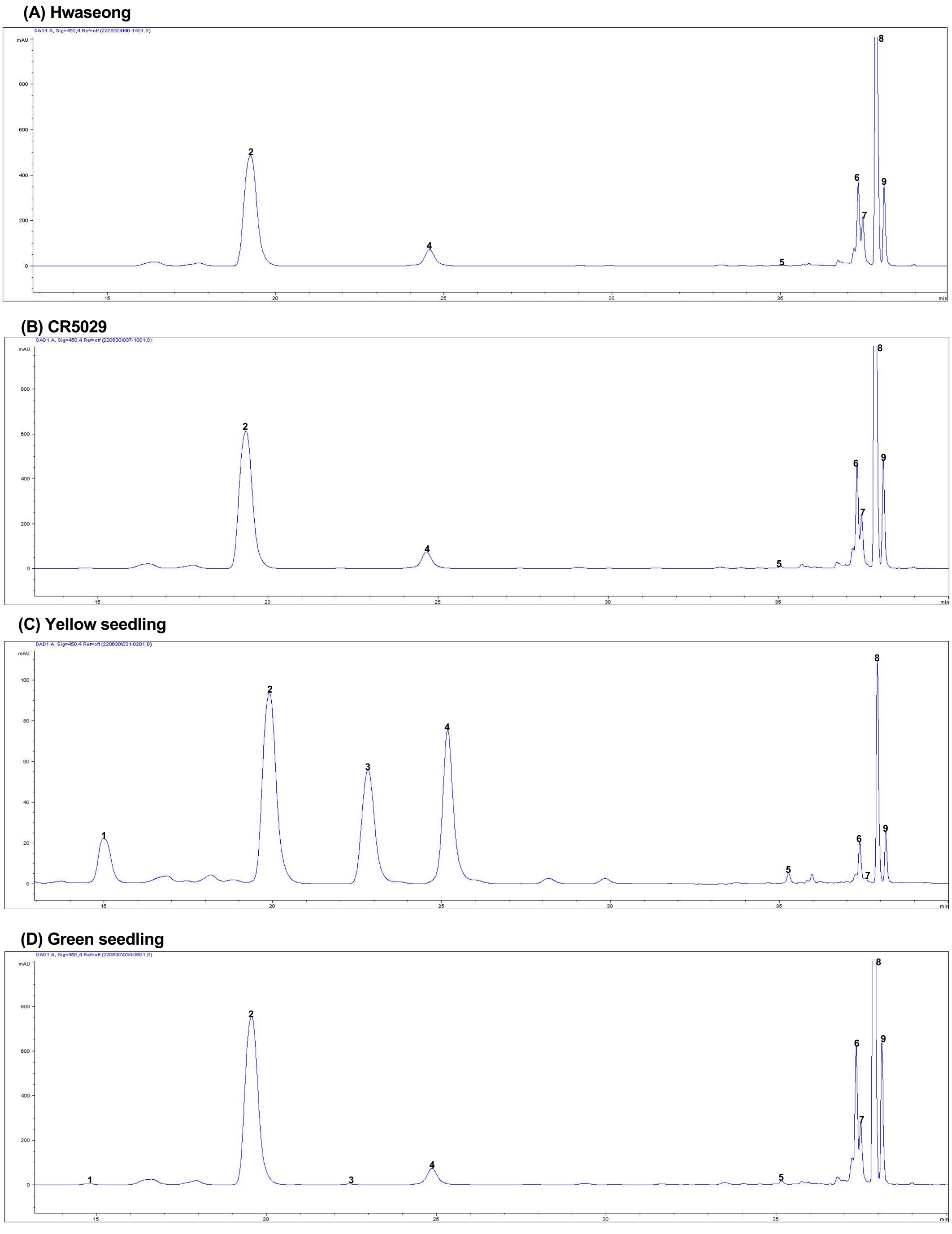

Figure S3. HPLC carotenoids chromatogram obtained from leaves of (**A**)Hwaseong, (**B**)CR5029, (**C**)Yellow seedling, and (**D**)Green seedling. Peak: 1. Antheraxanthin; 2. Lutein; 3. Zeaxanthin; 4. trans-β-Apo-8′-carotenal (internal standard); 5. β-Cryptoxanthin; 6. 13Z-β-Carotene; 7. α-Carotene; 8. E-β-Carotene; 9. 9Z-β-Carotene

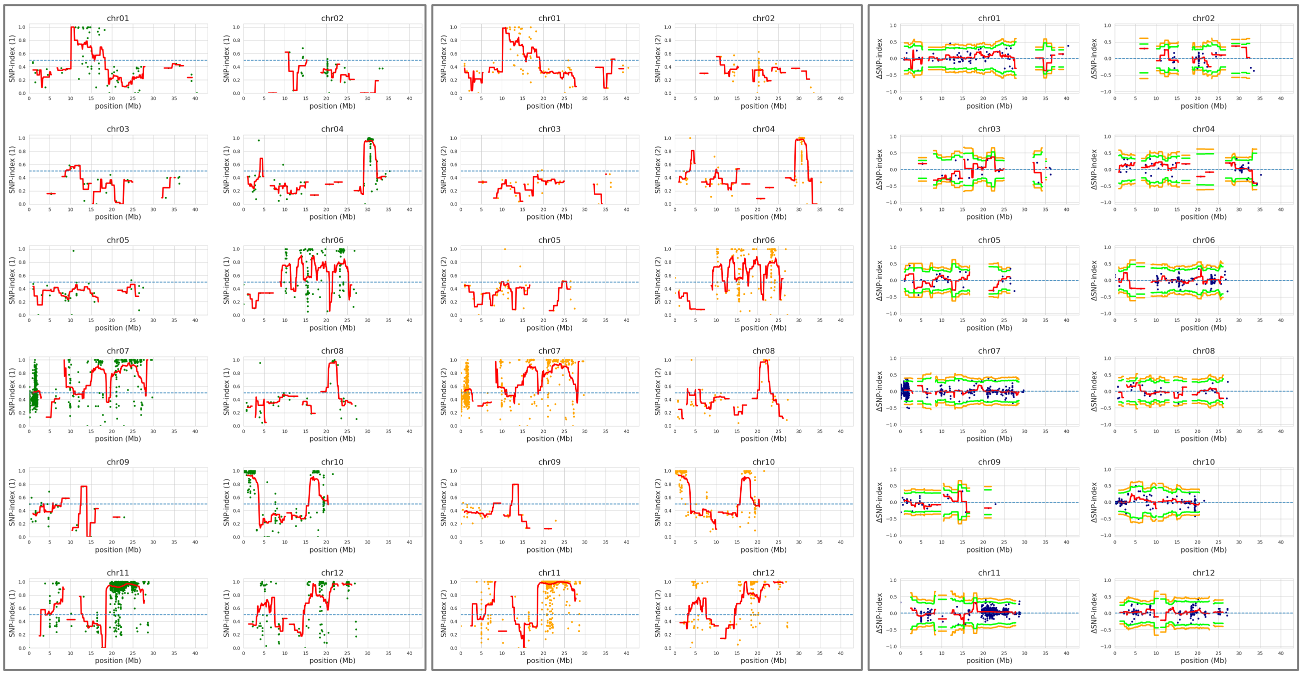

Figure S4. QTL-seq analysis to identify locus associated with the yellow seedling phenotype. CR5029 was used for parental reference sequence in QTL-seq analysis. Single nucleotide polymorphism (SNP)-index plot of GS bulk (green) and YS bulk (orange), and delta-SNP index (blue) were shown. Red line indicates mean of SNP-indices, and orange and green line indicate mean of 99% and 95% confidence interval of simulated delta SNP-indices (p99 and p95), respectively.

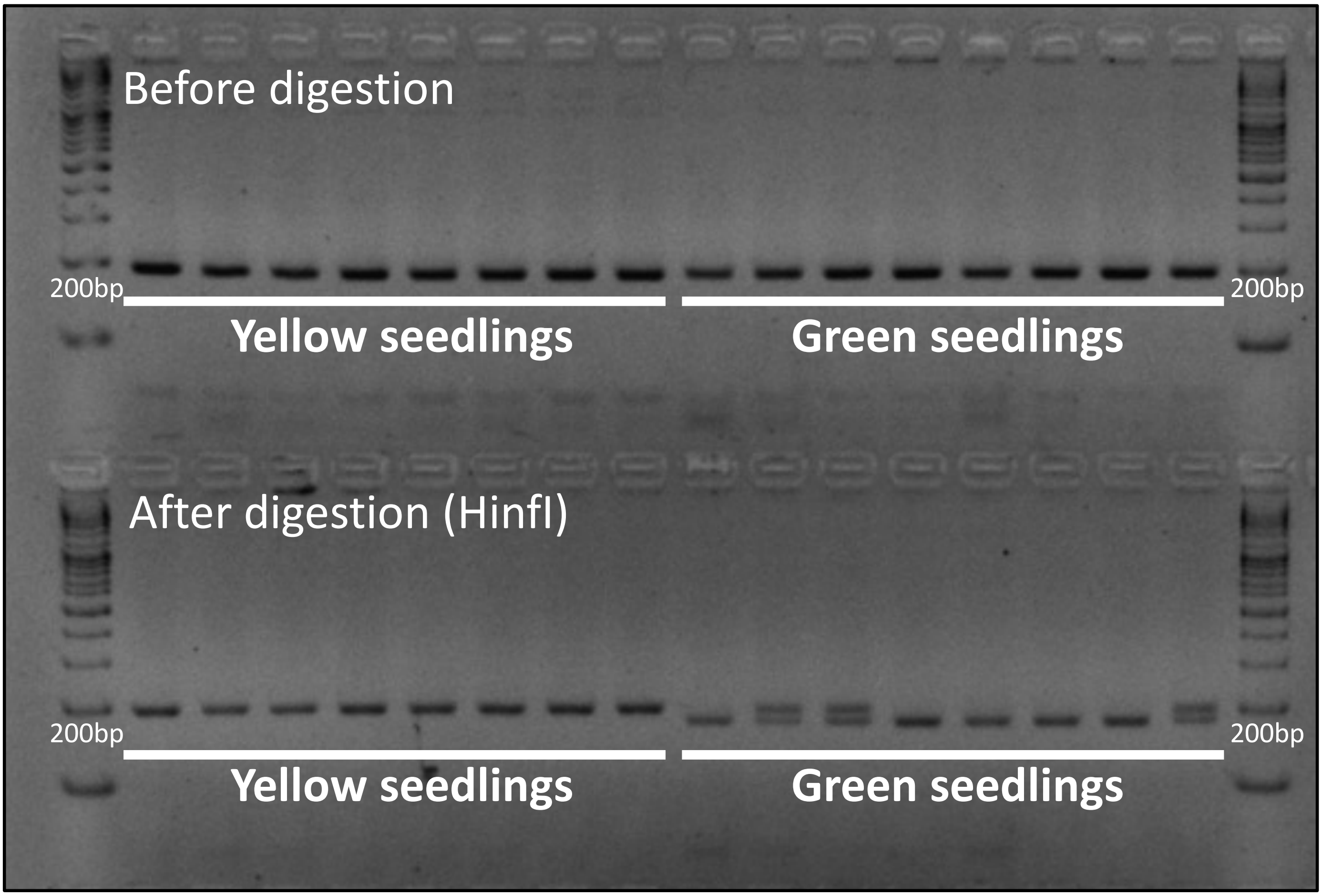

Figure S5. PCR amplicons of ELL_CAPs marker before and after digestion with restriction enzyme HinfI. Amplicons were separated on 3% Agarose gel stained with StaySafe Nucleic Acid Gel Stain (RBC).
